# Supplementary material for: The RNA-binding protein YBX1 regulates epidermal progenitors at a posttranscriptional level
Source: Nat Commun. 2018 Apr 30;9:1734. doi: 10.1038/s41467-018-04092-0 (PMC5928080; doi:10.1038/s41467-018-04092-0)
Supplement: Supplementary file 1 — Supplementary Information [file 41467_2018_4092_MOESM1_ESM.pdf]

Supplementary Information

The RNA-binding protein YBX1 regulates epidermal progenitors at a posttranscriptional level.

Kwon et al.

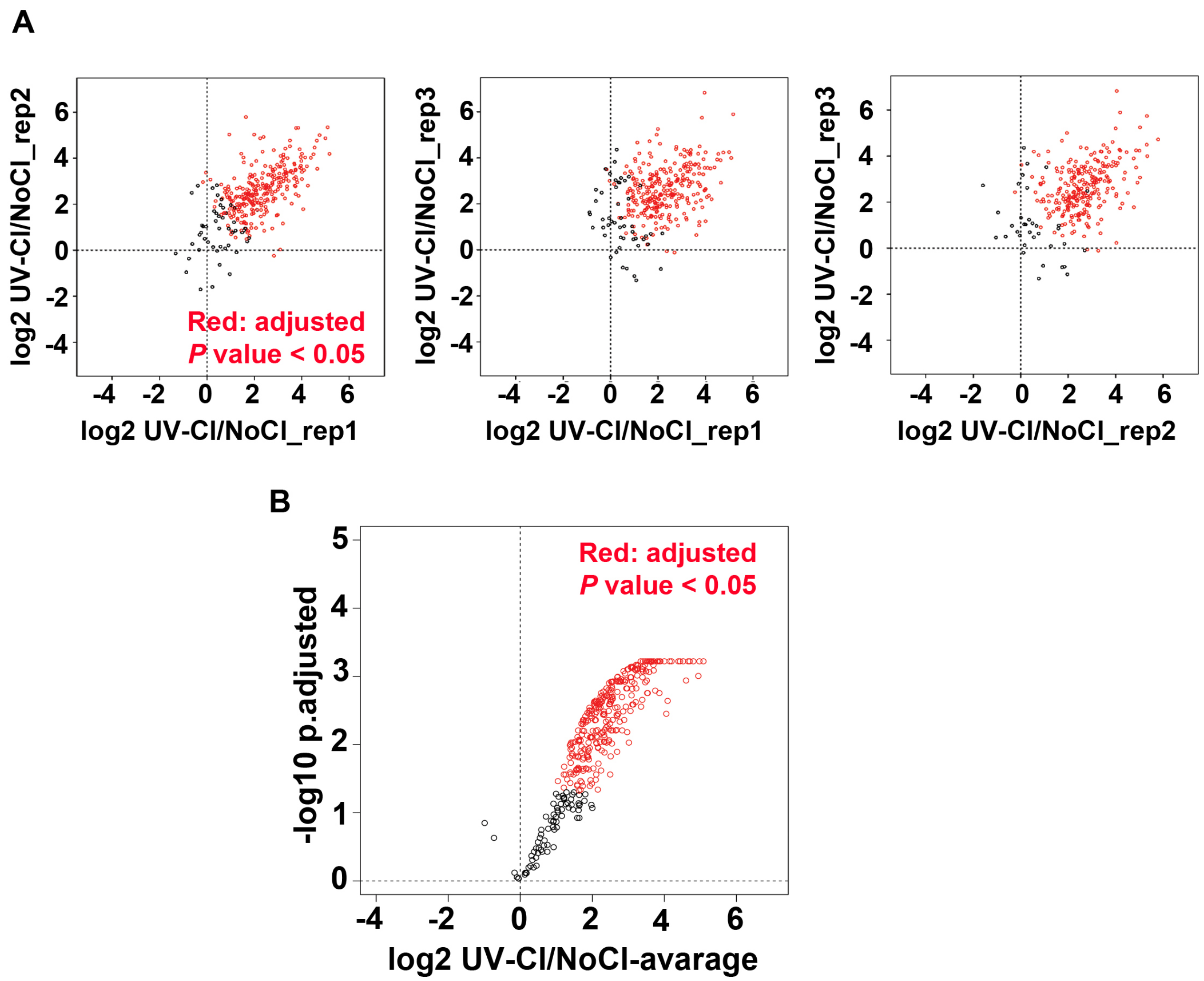

Supplementary Figure 1. Reproducibility and donor-related variability of mRNA interactome capture

(A) Scatter plots comparing the proteins enrichment in cross-linked (UV-Cl) versus non cross-linked (NoCl) samples. LC-MS intensities from three biological replicates (each one prepared from pooled primary HKC cultures derived from different donors) were plotted against each other. Each dot represents a single polypeptide. Red dots represent enriched proteins ($p < 0.05$, analyzed using Linear Models for Microarray Data (LIMMA)).

(B) Volcano plot displaying the average $\log_2$ ratios of UV-CI versus NoCI for all proteins quantified in at least two out of the three biological replicates plotted against their adjusted p values. Each dot represents a single protein and dots in red are displaying proteins with a fold change significantly ($p < 0.05$, analyzed using Linear Models for Microarray Data (LIMMA)) different from 0.

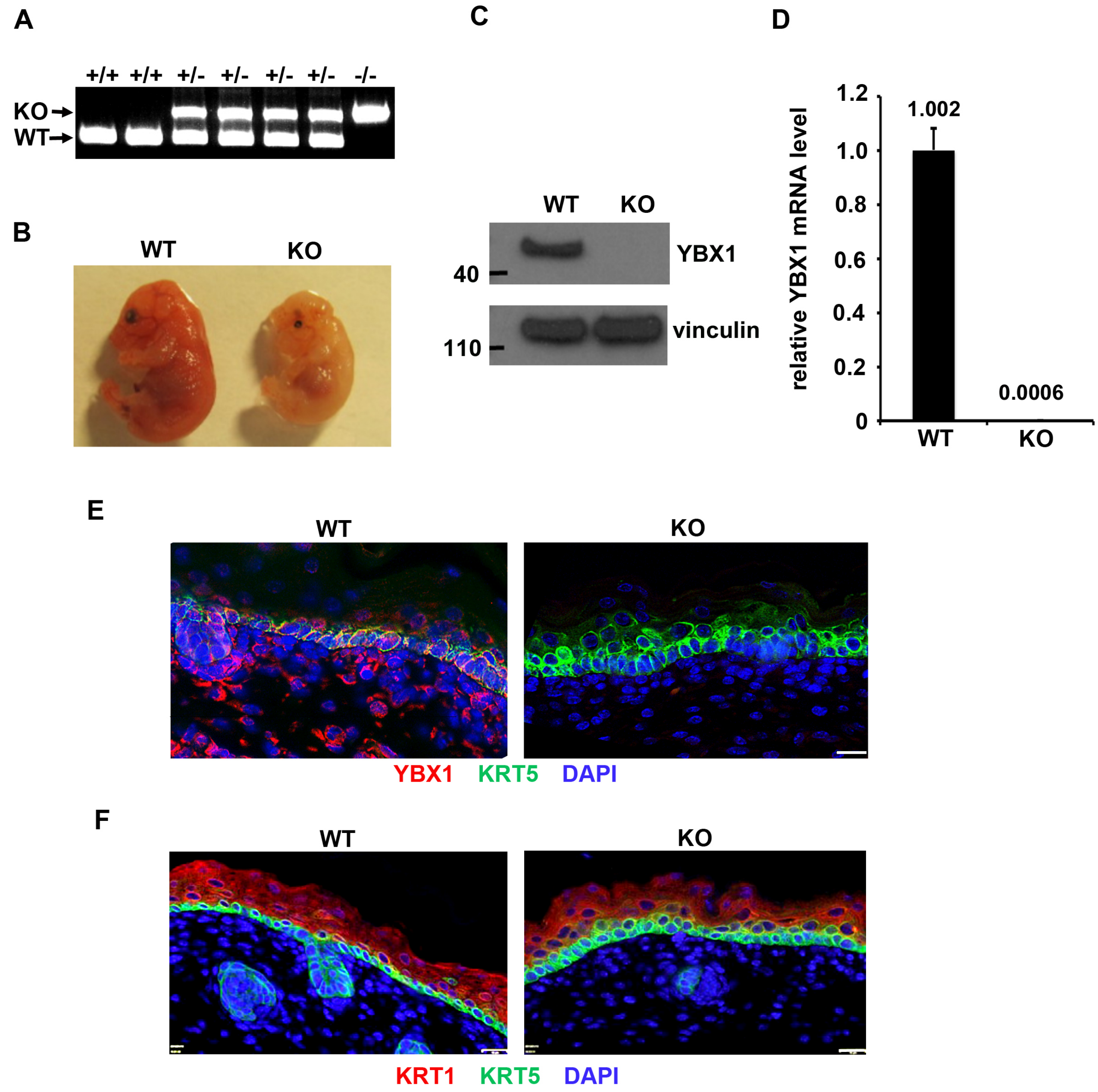

Supplementary Figure 2. Phenotypic changes in the YBX1 knockout mice

(A) PCR genotyping of seven E16 embryos. Bands of wild type (WT) and YBX1 knockout (KO) are shown.

(B) WT and YBX1 KO embryos at E16 stage of development. YBX1 KO embryos exhibit moderate growth retardation.

(C, D) YBX1 expression levels (protein in (C) and mRNA in (D)) were measured in mouse primary keratinocytes isolated from YBX WT and KO embryos at E16. Vinculin was used for loading control in the Western blot (C) and m36B4 in the qRT-PCR (D) experiments.

(E) Mouse epidermis from WT and KO E16 embryos was stained for YBX1 (red) and for the basal layer marker KRT5 (green), while DAPI staining (blue) was used to visualize the nuclei. Scale bar is 16 μm.

(F) Mouse epidermis from WT and KO E16 embryos was stained for the basal layer marker KRT5 (green) and the suprabasal differentiation marker KRT1 (red). DAPI staining was used to visualize the nuclei. Scale bar is 16 μm.

Images shown in E and F are representative of n=4.

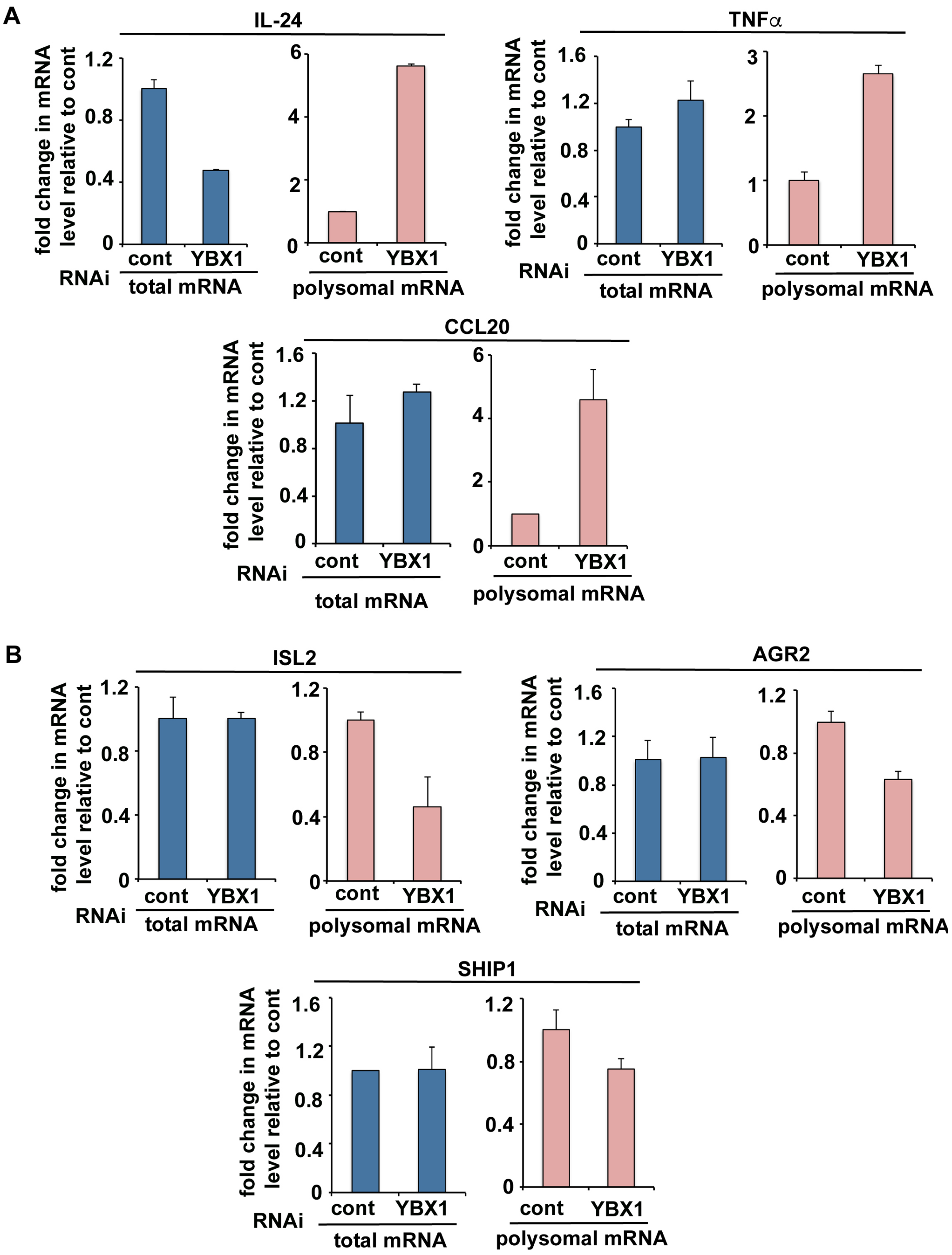

Supplementary Figure 3. YBX1 depletion affects translation of downstream target mRNAs

(A) Polysome associated mRNAs, <u>up regulated</u> upon YBX1 depletion: relative IL-24, TNFα and CCL20 mRNA levels in control and YBX1 siRNA transfected HKC as measured by quantitative

qRT-PCR of total RNA or polysomal fractionated RNA after sucrose gradient centrifugation of total RNA. 36B4 mRNA was used as an internal control. Error bars represent mean SD, n=3.

(B) Polysomal mRNAs, down modulated in YBX1 KD cells: The experiments were performed as in (A) and relative mRNA levels of ISL2, AGR2 and SHIP1 were analyzed by qRT-PCR using 36B4 as a control. Error bars represent mean SD, n=3.

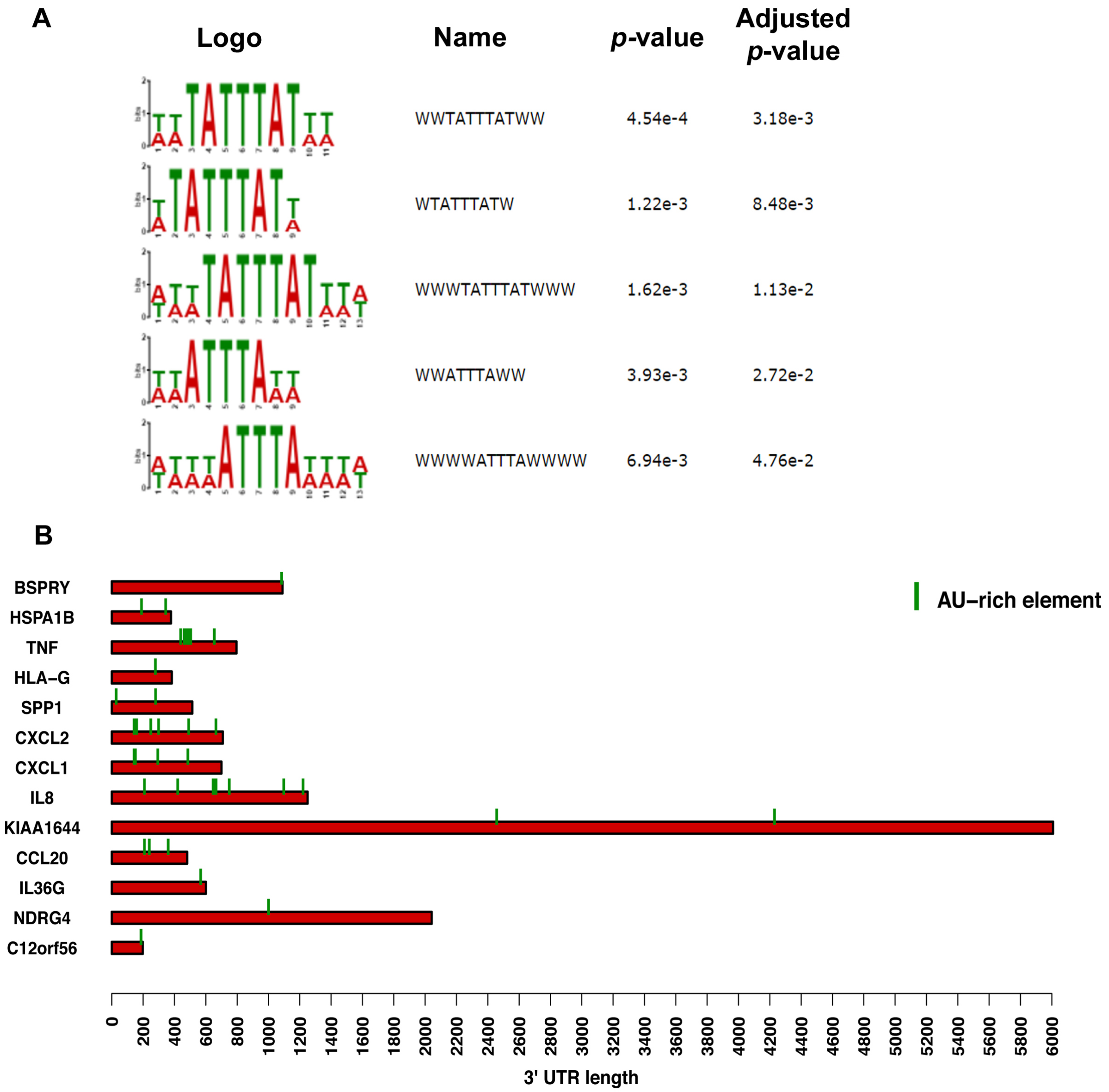

Supplementary Figure 4. AU-rich elements in the 3’UTRs of YBX1 targets

(A) Analysis of Motif Enrichment (AME) [1] showed a significant enrichment for 5 out of 6 ARE motifs on the 3’UTRs sequence of 13 out of the 23 down-regulated transcripts from Table S3. ARE motifs position-specific scoring matrices were obtained from Atlas of UTR Regulatory Activity (AURA) [2].

(B) ARE motifs positional matching on 13 out of the 23 3’UTRs in Table S3, according to AURAlight cis elements mapping [2].

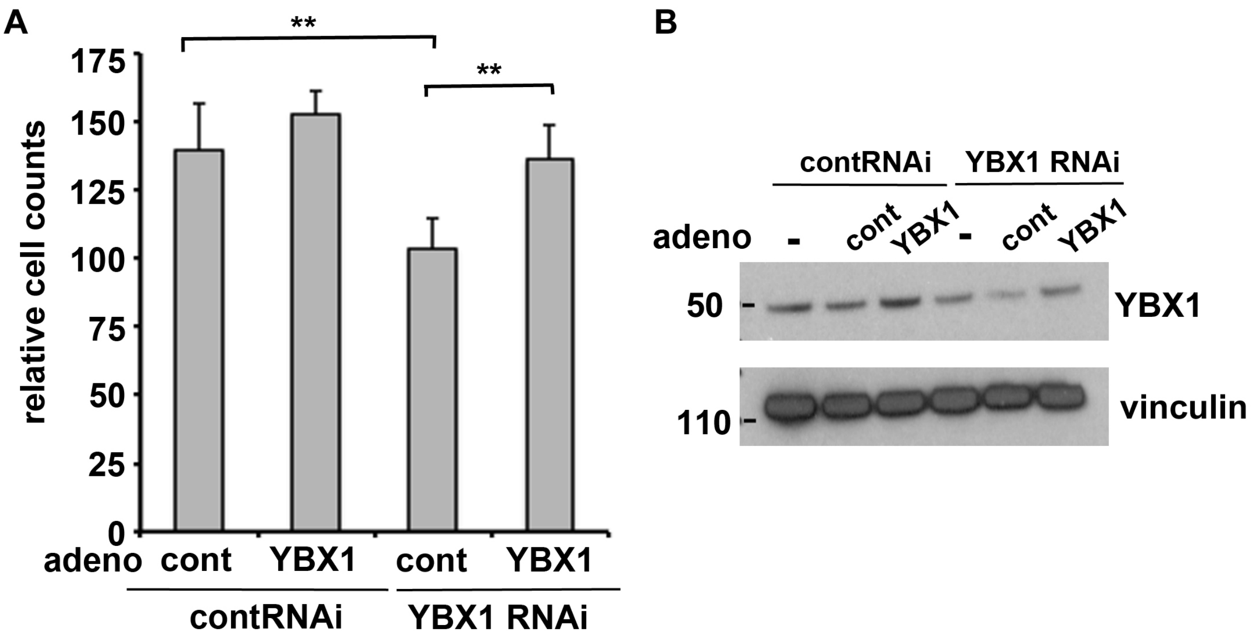

Supplementary Figure 5. Effects of RNAi mediated depletion of YBX1 on cell numbers

(A) Primary cultures of human keratinocytes were transiently transfected with control or YBX1 specific siRNAs (custom designed as explained in Experimental Procedures) and with control or RNAi resistant YBX1 adenoviruses. Cells from each well of a 6 well culture plate were harvested, re-suspended in 1ml of medium, 10 μl were loaded in a haemocytometer and relative counts per 1 μl are presented as bar graphs. Error bars represent mean SD, n=3, **$p<0.01$, unpaired t-test.

(B) Western blot analysis was used to detect levels of expression of YBX1 in the cultures used in (A).

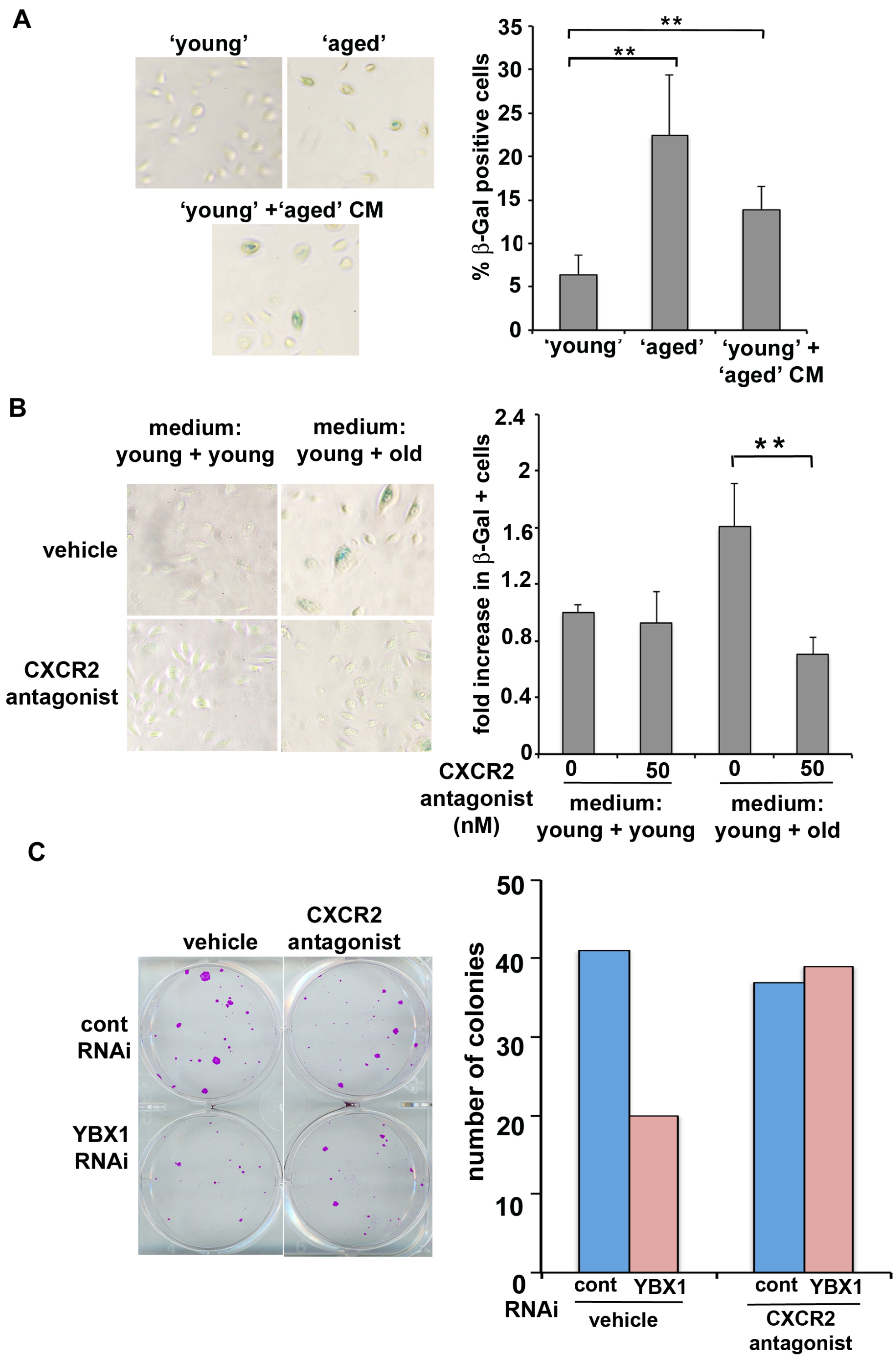

Supplementary Figure 6. Cytokine dependent senescence in human keratinocytes

(A) Primary human keratinocytes were isolated from 'young' (18 or 30 years old) and 'aged' (68 and 74 years old) donors and placed in culture. The 'young' cells were grown either in their own

culture medium, ‘young medium’, or in ‘young medium’, mixed with ‘aged medium’ (previously conditioned by the aged cultures). Cultures were stained with SA-β-Gal. Total and SA-β-Gal positive cells were counted under light microscopy (x40). Result shows percentage of SA-β-Gal positive cells in each group (*, $p<0.05$, unpaired t-test). Error bars represent mean SD, n=6.

(B) Human keratinocytes from the ‘young’ donors were grown in control medium or in fresh medium mixed with medium from the ‘aged’ donors, treated with either vehicle or the CXCR2 antagonist and stained for SA-β-gal. Total and SA-β-Gal positive cells were counted under light microscopy (x40). Results show fold change in SA β-gal positive cells relative to untreated ‘young’ cultures, (**, $p<0.01$, unpaired t-test). Error bars represent mean SD, n=5.

(C) Primary human keratinocytes transfected with control or YBX1 siRNA were seeded at clonal density (100 cells per well) over Swiss3T3 cells feeder layer and treated with vehicle control or the small molecule CXCR2 antagonist. 10 days later the cultures were fixed, the colonies were stained with SRB and counted using ImageJ.

Supplementary Fig. 7

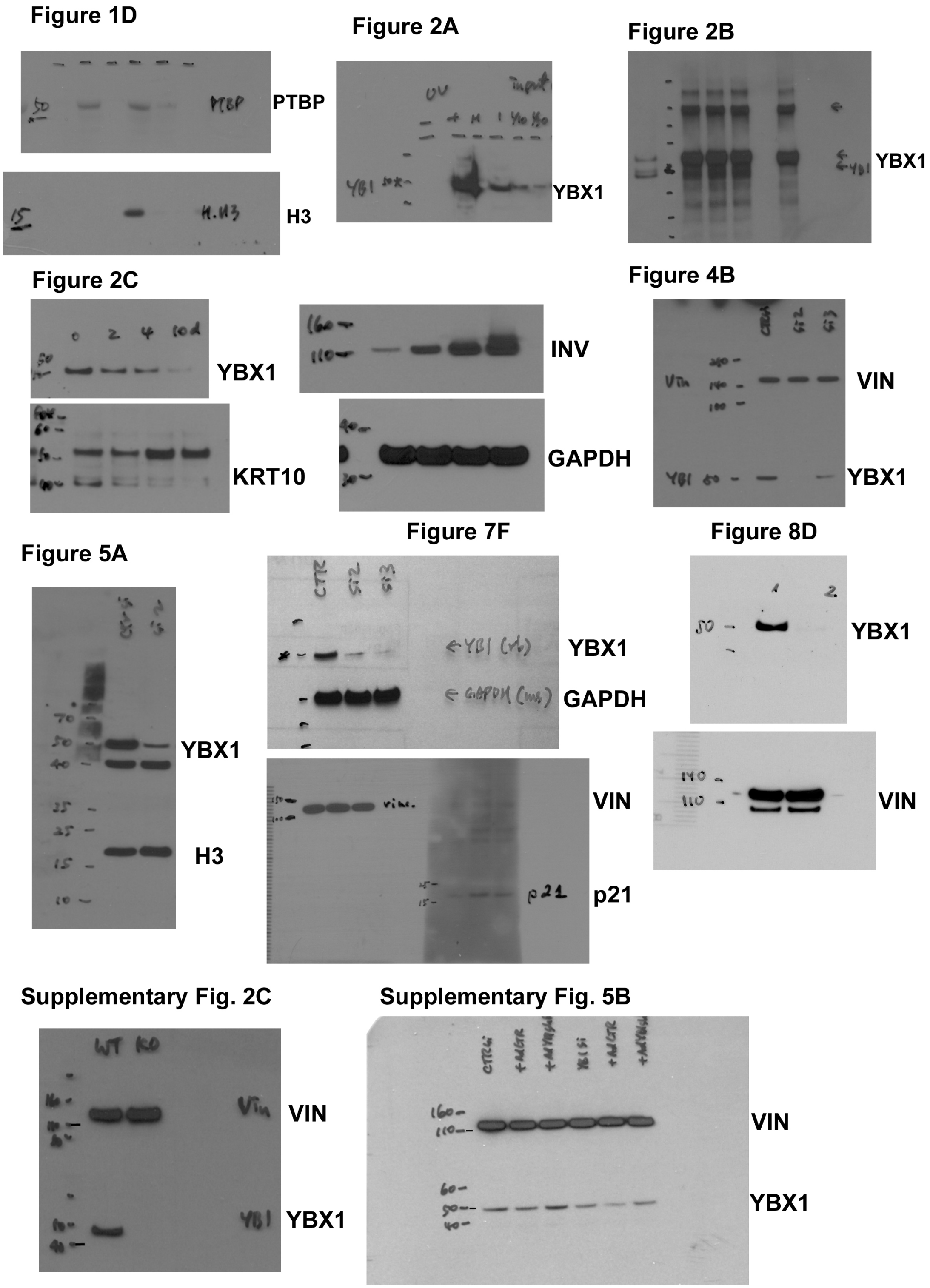

Supplementary Figure 7. Uncropped scans of the Western blots shown in this paper

Gene name	Forward(5')	Reverse(5')
Specific Primers for qRT-PCR		
mouse		
mYBX1	GGGATCGGAAAGCGCTCCTG	CTTGCTCTCCTGCACCCTGG
mKRT10	TGTGAATGTGGAAATGAACG	GAAGAGCAAGGAACTCACCA
m36B4	AGGGTGTCCGCAACGTGGCCAGTG	AGCTGCACATCACTCAGAATTTCA
mYWHAZ	GAAAAGTTCTTGATCCCCAATGC	TGTGACTGGTCCACAATTCCTT
mIL-8	CAAGGCTGGTCCATGCTCC	TGCTATCACTTCCTTTCTGTTGC
mCXCL1	CTGGGATTCACCTCAAGAACATC	CAGGGTCAAGGCAAGCCTC
m18S	TTGACGGAAGGGCACCACCAG	CTCCTTAATGTCACGCACGATTTC
human		
YBX1	TCGCCAAAGACAGCCTAGAGA	TCTGCGTCGGTAATTGAAGTTG
36B4	GCAATGTTGCCAGTGTCTGT	GCCTTGACCTTTTCAGCAAG
YWHAZ	ACTTTTGGTACATTGTGGCTTCAA	CCGCCAGGACAAACCAGTAT
KRT1	GTTCCAGCGTGAGGTTTGTT	TAAGGCTGGGACAAATCGAC
KRT10	GAAAAGCATGGCAACTCACA	CATCCTGCTTCAGATCGACA
Involucrin	GGCCCTCAGATCGTCTCATA	CACCCTCACCCCATTAAAGA
CXCL1	AACCGAAGTCATAGCCACAC	GTTGGATTTGTCACTGTTCAGC
CXCL2	TGCCAGTGCTTGCAGAC	TCTTAACCATGGGCGATGC
IL8	CTTGGCAGCCTTCCTGATTT	TTCTTTAGCACTCCTTGGCAAAA
IL24	AAGCAGATCCTCAATAAACATTTC	ACCAAGGGAAAGGGATGATG
CCL20	CCAAGAGTTTGCTCCTGGCT	TGCTTGCTGCTTCTGATTCG
TNFa	ATCTTCTCGAACCCCGAGTGA	GGGTTTGCTACAACATGGGC
Specific Primers for RIP		
YBX1	TCGCCAAAGACAGCCTAGAGA	TCTGCGTCGGTAATTGAAGTTG
CXCL1	AACCGAAGTCATAGCCACAC	GTTGGATTTGTCACTGTTCAGC
CXCL2	TGCCAGTGCTTGCAGAC	TCTTAACCATGGGCGATGC
IL-8	CTTGGCAGCCTTCCTGATTT	TTCTTTAGCACTCCTTGGCAAAA
ND4L	TCCTCCCTACTATGCCTAGAAGGA	CTTCGCAGGCGGCAAA
ND1	CCCTAAAACCCGCCACATCT	GGCTAGAATAAATAGGAGGCCTAGGT
ND4	TCACAACACCCTAGGCTCACTAA	GGGAGTCATAAGTGGAGTCCGT
ATP8	GCCCCAACTAAATACTACCGTATGG	GGCTTTGGTGAGGGAGGTA
Specific Primers for mRNA interactome		
18s	GAAACTGCGAATGGCTCATTAAA	CACAGTTATCCAAGTGGGAGAGG
β-actin	CGCGAGAAGATGACCCAGAT	TCACCGGAGTCCATCACGAT
HPRT	CCTGGCGTCGTGATTAGTGAT	AGACGTTCAGTCCTGTCCATAA
Luciferase	GAATTTGCAGCATATCTTGAACCAT	GGATTTCACGAGGCCATGATAA

Supplementary Table 1. List of specific primers used in the study

Supplementary references:

1. McLeay RC, Bailey TL. Motif Enrichment Analysis: a unified framework and an evaluation on ChIP data. *BMC Bioinformatics* **11**, 165 (2010).

2. Dassi E*, et al.* AURA 2: Empowering discovery of post-transcriptional networks. *Translation (Austin)* **2**, e27738 (2014).
