# Supplementary material for: The RNA-binding protein YBX1 regulates epidermal progenitors at a posttranscriptional level
Source: Nat Commun. 2018 Apr 30;9:1734. doi: 10.1038/s41467-018-04092-0 (PMC5928080; doi:10.1038/s41467-018-04092-0)
Supplement: Supplementary file 3 — Description of Additional Supplementary Files [file 41467_2018_4092_MOESM3_ESM.docx]

**Description of Additional Supplementary Files**

File Name: Supplementary Data 1

Description: mRNA interacting proteins in proliferating epidermal progenitors

Three independent pooled cultures of primary human keratinocytes isolated from different donors were used to capture mRNA interacting proteins. Samples were analysed on a 240 min. gradient on the LC/MS-MS system (Thermo Fisher LTQ Velos Pro). The raw data was analysed with MaxQuant (v. 1.4.1.2) and further processed using Perseus (v.1.4.0.11). Linear Models for Microarray Data (LIMMA) test was performed on the replicates and a p.adjusted value was only calculated when a ratio in 2 out of 3 samples was present.

File Name: Supplementary Data 2

Description: Classical RBPs highly expressed in proliferating epidermal progenitors and down-modulated upon keratinocytes differentiation

RBPs identified by interactome capture experiments and annotated as modulators of RNA biology utilizing GO were further analysed for their expression levels during keratinocyte commitment to differentiation using a publically available dataset. 61 of these annotated RBPs with high level of expression in proliferating cells (greater than 30 FPKM) were suppressed upon keratinocyte commitment to differentiation. Among those YBX1 is highlighted in yellow.

File Name: Supplementary Data 3

Description: Changes in total mRNAs upon YBX1 siRNA mediated KD

File Name: Supplementary Data 4

Description: Transcriptome (RNA-seq) analysis of total and polysome associated mRNA from primary human keratinocytes transfected with control or YBX1 siRNA

Translational targets of YBX1 were identified based on control versus two biological replicates of YBX1 siRNA transfected samples. Highlighted are transcripts that were equally expressed within total RNA populations of control and YBX1 siRNA cells but were selectively modulated in the polysomal mRNA fractions of YBX1 depleted cells.

File Name: Supplementary Data 5

Description: Pathways and functional analysis was performed using PANTHER software provided by the Gene Ontology Consortium: <http://geneontology.org>
